# Novel Avian Influenza H7N3 Strain Outbreak, British Columbia

**DOI:** 10.3201/eid1012.040743

**Published:** 2004-12

**Authors:** Martin Hirst, Caroline R. Astell, Malachi Griffith, Shaun M. Coughlin, Michelle Moksa, Thomas Zeng, Duane E. Smailus, Robert A. Holt, Steven Jones, Marco A. Marra, Martin Petric, Mel Krajden, David Lawrence, Annie Mak, Ron Chow, Danuta M. Skowronski, S. Aleina Tweed, SweeHan Goh, Robert C. Brunham, John Robinson, Victoria Bowes, Ken Sojonky, Sean K. Byrne, Yan Li, Darwyn Kobasa, Tim Booth, Mark Paetzel

**Affiliations:** * British Columbia Cancer Agency (BCCA) Genome Sciences Centre, Vancouver, British Columbia, Canada;; † British Columbia Centre for Disease Control and University of British Columbia Centre for Disease Control, Vancouver, British Columbia, Canada;; ‡ Ministry of Agriculture, Abbotsford, British Columbia, Canada;; § Canadian Centre for Human and Animal Health, Winnipeg, Manitoba, Canada;; ¶ Simon Fraser University, Burnaby, British Columbia, Canada

**Keywords:** avian influenza, genomic sequence, recombination, Homo sapiens, Virulence

## Abstract

Genome sequences of chicken (low pathogenic avian influenza [LPAI] and highly pathogenic avian influenza [HPAI]) and human isolates from a 2004 outbreak of H7N3 avian influenza in Canada showed a novel insertion in the HA0 cleavage site of the human and HPAI isolate. This insertion likely occurred by recombination between the hemagglutination and matrix genes in the LPAI virus.

Highly pathogenic avian influenza (HPAI) viruses cause systemic disease in poultry, which is associated with rapid death and a case-fatality ratio approaching 100%. To date, only H5 and H7 subtypes have shown this virulence, although not all of these subtypes are HPAI. HPAI viruses are not normally present in wild bird populations but arise from low pathogenic avian influenza (LPAI) viruses introduced into poultry flocks from wild birds (*1**,**2*).

The hemagglutinin gene plays a key role in defining virulence in avian influenza (AI). The hemagglutinin glycoprotein is produced as a precursor, HA0, which requires posttranslational cleavage by host proteases before infectious virus particles can be produced (*3*). Cleavage of the HA0 precursor in LPAI viruses is catalyzed only by trypsin and trypsinlike host proteases restricting virus replication to locations where these proteases are found, namely, respiratory and intestinal tracts. In contrast, HA0 cleavage in HPAI viruses is mediated by a poorly defined protease(s) that appears to be a proprotein-processing subtilisin-related endoproteases (*4*). The ubiquitous nature of these protease(s) enables the HPAI virus to replicate systemically, damaging vital organs and tissues, leading to disease and death (*3*).

All HPAI viruses encode a HA0 protein having a motif of multiple basic amino acids (R and K) flanking the cleavage site. In contrast, LPAI viruses have two basic amino acids at positions –1 and –4 from the cleavage site for H5 and at positions –1 and –3 for the H7 subtype. An increase in basic residues near the cleavage site, either as a result of nucleotide insertion or substitution, allows the HA0 precursor to be cleaved by ubiquitous host proteases (*5*).

Since 1996, several instances of AI viruses infecting humans have been reported; some of these cases have been fatal (*6*). Here we describe the sequence of avian and human LPAI and HPAI isolates obtained from the AI outbreak in the Fraser Valley of British Columbia in 2004. Avian HPAI and human virus isolates contain an insert that does not conform to the consensus sequence suggested to be the prerequisite for all HPAI viruses (*7*), and further analysis shows the insertion is the result of nonhomologous recombination between the hemagglutinin and matrix genes of the virus. Both human isolates have mutated since the original recombination event, and one of the two is likely not highly pathogenic in chickens. We also provide a homology model for HA0 of the HPAI human isolate and show that in addition to adding basic residues, the H1 insertion likely increases accessibility to the protease cleavage site.

## The Study

All methods and materials, including supplementary data, are available (Appendix). At the index farm, two flocks were maintained in adjacent barns. Decrease in appetite and slightly increased death rate were noted in the older flock, followed by a dramatic increase in death rate (25% in 48 hours) in the younger flock. Influenza A virus was isolated from both flocks: A/Chicken/Canada/AVFV1/04 (AVFV1) from the older and A/Chicken/Canada/AVFV2/04 (AVFV2) from the younger. All birds on this farm were culled. However, the virus spread, resulting in a Canadian Food Inspection Agency order to kill all 19 million domestic birds in the Fraser Valley.

In two workers involved in the depopulation, symptoms developed, including conjunctivitis, headache, and coryza, 1–3 days after direct exposure of the eye to poultry tissue on infected farms. Influenza A (H7N3) was isolated from both persons (*8*). The genomes of the viral isolate were sequenced to determine whether genetic changes were associated with increased pathogenicity and to assess whether the virus had acquired human influenza A genes. Four isolates were sequenced: two poultry viruses, AVFV1 and AVFV2, and the two human isolates A/Canada/444/04 (human) (Hu444) and A/Canada/504/04 (human) (Hu504).

**Figure 1:**
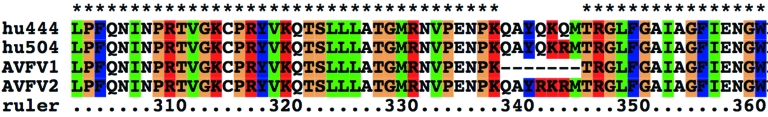
Alignment of the hemagglutinin cleavage region from four isolates of Fraser Valley H7N3 virus. A/Chicken/Canada/AVFV1/04 is designated AVFV1; A/Chicken/Canada/AVFV2/04 is designated AVFV2; A/Canada/444/04 (human) is Hu444, and A/Canada/504/04 (human) is Hu504). A 7–amino-acid (aa) insertion associated with the AVFV2 isolate and both human isolates is shown at aa 338.

**Figure A1:**
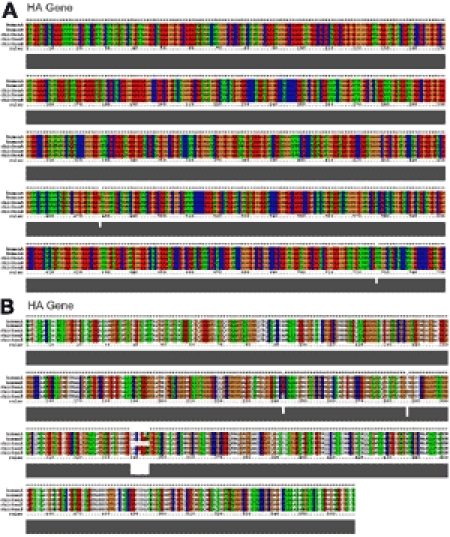
Complete nucleotide (A) and protein (B) alignments from the 5 isolates sequenced in this study. The alignments were generated by ClustalX (1.82) (http://www.embl.de/~chenna/clustal/darwin/). [A/Canada/AVFV1/04 (environmental) is designated as chicken_A; A/Chicken/Canada/AVFV2/04 is designated as chicken_B; A/Canada/444/04 (human) is human_A and A/Canada/504/04 (human) is human_B. Full sequenced segments for chicken_D and chicken_E, not described in the text, are included for completeness.

Consensus sequences for the eight genomic segments isolated from each of the four independent viral genomes are deposited in GenBank. Accession numbers are listed in the Table A1 and ClustalW lineups of the complete nucleotide and protein sequences are available (Figure A1, A and B, respectively; available at insert B). Sequences for all 32 genes (and their encoded proteins) are highly related to previously determined sequences (BLASTN identities 93%–98% and BLASTP identities 98%–100%) (Table A1) and do not suggest the presence of human influenza A genes. However, hemagglutinin (HA) genes in three of the Fraser Valley isolates (AVFV2, Hu444, and Hu504) have a 21-nucleotide (nt) (7 amino acid [aa]) insertion in the HA gene (protein) immediately upstream of the HA0 cleavage site relative to the most closely related H7N3 sequence. AVFV1 lacked this insertion. Although both human isolates contained the insertion, amino acid changes within the inserted sequence were observed compared with the avian AVFV2 sequence, indicating that sequence drift occurred after the initial insertion event. Figure 1 is an alignment of the HA0 cleavage region of all four isolates. An examination of the sequences indicates that the insertion of the 21 nt likely occurred once (QAYRKRM–AVFV2) and that the sequence has subsequently mutated to QAYQKRM and QAYQKQM in human isolates Hu504 and Hu444, respectively.

When the sequence of the AVFV2 virus from the FV outbreak was analyzed, the 21-base insert matched perfectly with a region from the influenza matrix (M) gene. For the two human isolates, the match is 20/21 nt (Hu504) and 19/21 nt (Hu444), indicating this sequence changed over a short period, which is consistent with a high rate of mutation in influenza viruses.

The two avian isolates were tested for pathogenicity by the National Centre for Foreign Animal Diseases by using standard in vivo testing in chicks (http://www.inspection.gc.ca/english/anima/heasan/disemala/avflu/avflufse.shtml). AVFV1 was not pathogenic, whereas AVFV2 is highly pathogenic. On the basis of the consensus sequence for highly pathogenic H7 viruses, we can predict that the Hu504 isolate is likely highly pathogenic in chickens while the Hu444 isolate is likely not pathogenic. These tests are currently under way in another laboratory.

**Figure A2:**
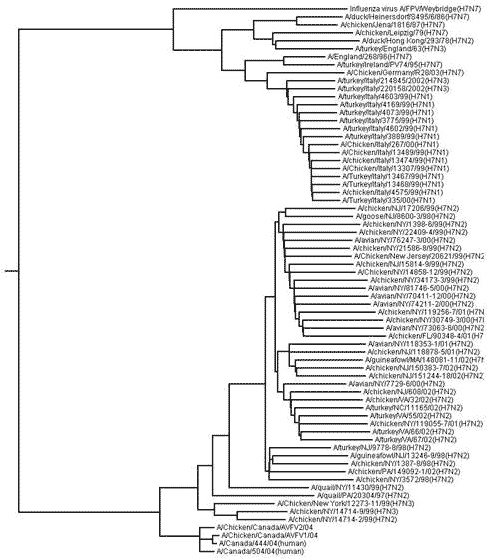
Phylogenetic tree of isolates described in the text with 65 full-length H7 HA gene segments present in GenBank. The tree was generated with nucleotide sequences by the neighbor-joining method in Bonsai (1.1.4.) (http://calliope.gs.washington.edu/software/). Corrected distance estimate is indicated by the scale bar. The H7N3 avian influenza isolates reported in this article are at the bottom of the tree.

To further characterize the hemagglutinin gene from the Fraser Valley HPAI isolate, a phylogenetic tree was generated by using an alignment of 65 full-length H7 HA sequences obtained from GenBank (Figure A2, available at insert C). With the exception of a single isolate, A/duck/Hong Kong/293/78, the sequences clustered into two distinct sublineages based on location of origin; North American or European. Isolates within the North American sublineage were further divided on a 24-nt deletion (beginning at nt 710) not found in the Fraser Valley isolates. This deletion, first reported in LPAI H7N2 isolates originating from American live bird markets (*9*), lies within the receptor-binding site for influenza viruses and is thought to compensate for a concurrent NA stalk deletion (*10*). Consistent with the absence of this deletion in our isolates, no deletion was observed in the NA stalk region of the four Fraser Valley isolates.

The insertion in the HA protein sequence from the Fraser Valley isolates does not conform to the consensus motif (R-X-R/K-R*-G-L-F) for an HA1–HA2 connecting peptide in HPAI viruses because a threonine is at the -2 position. However, the AVFV2 and Hu504 insertions do conform to a minimum cleavage recognition sequence associated with HPAI viruses (R-X-X-R*G) (*5*). Thus, the insertion sequences for these two Fraser Valley isolates are expected to be cleaved by furinlike proteases. (They may also be susceptible to chymotrypsinlike enzymes because of the introduction of a tyrosine residue at –6 in the HPAI isolate and at –4 and –6 in the human isolates.) Hu444 is likely not pathogenic in chickens, which is consistent with previous observations that a basic amino acid at –4 is required for cleavage (*5*).

**Figure 2:**
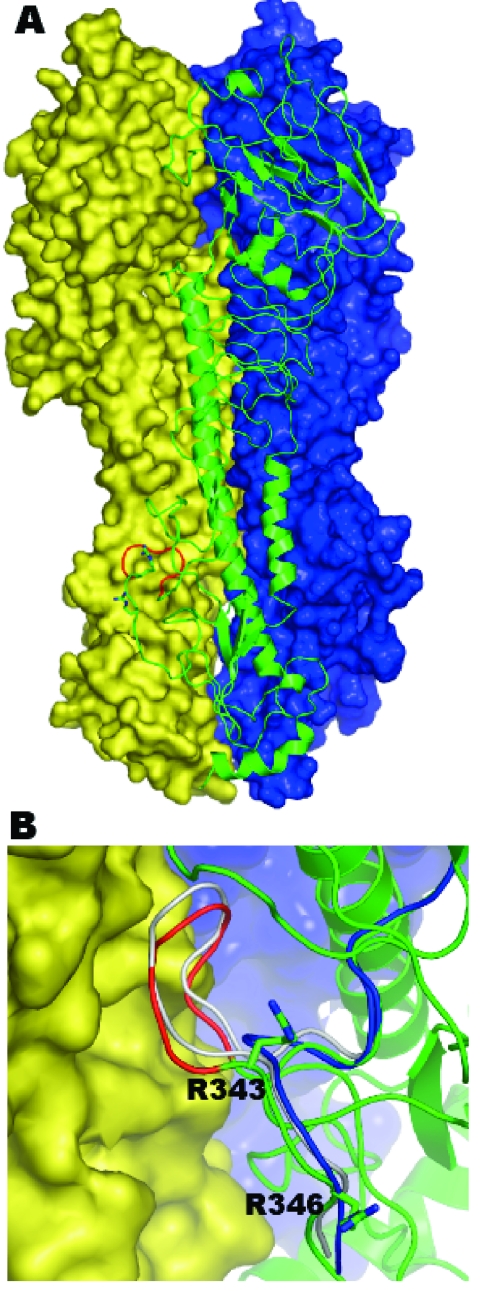
A homology model of the human A/Canada/504/04 (Hu504) hemagglutinin precursor (HA0) trimer based on the crystal structure of the human strain CV-1 HA0 (PDB: 1HA0) sequence identity 49.9%. A) Molecule A is shown as a green ribbon diagram; molecules B and C are shown in blue and yellow molecular surfaces, respectively. The 8–amino-acid (aa) sequence 335-342 (NPKQAYQK) is shown in red. B) A close up of this region located between molecules A (in green ribbon) and molecule C (in yellow surface). This 8-aa sequence forms a loop, which bumps into the adjoining molecule before energy minimization (gray). Shown in red is the loop after energy minimization, which results in the cleavage site's being pushed out slightly. Shown in blue is the corresponding region for the template structure (PDB code 1HA0). The side chains for arginine 343 and arginine 346 (–1 residue) are shown in stick form. (Since the preparation of this manuscript, the structure of an H7 HA protein has been reported [*12*]).

**Figure A3:**
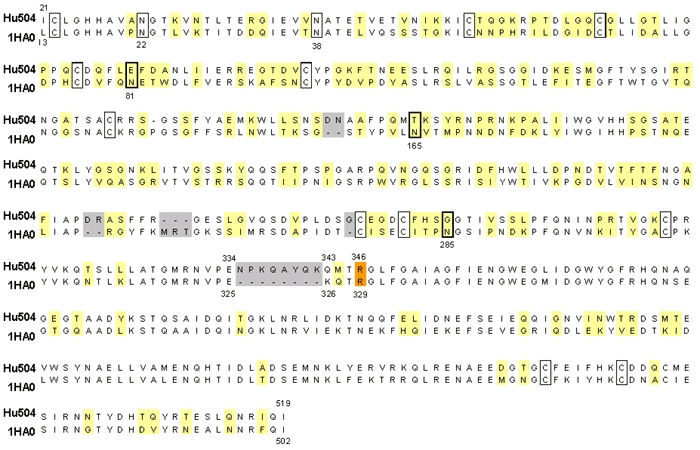
A structure-based sequence alignment of the influenza A virus hemagglutinin precursor protein from human A/Canada/504/04 (Hu504) with that of the hemagglutinin precursor protein (HA0) from influenza A virus, human strain CV-1 whose structure was solved by Wiley and colleagues in 1998 (PDBcode 1HAO) (*22*). The alignment shows 49.9% identity in sequence. Insertions and deletions are highlighted in grey and nonconservative mutations are shown in yellow. Conserved cysteines are boxed. The glycosylated aparagines which are conserved are boxed with thin lines; the glycosylated asparagines that are not conserved are boxed with thick lines.

Since the insertion sequence we observed in the HA gene is novel, we used molecular modeling to visualize the effect this insertion has on the HA0 protein. The model for the H7N3 hemagglutinin precursor is based on the 2.8 Å human H3 HA0 structure (*11*). From the structure-base sequence alignment (Figure A3) and resulting homology model (Figure 2) the 7-residue insertion extends out in a loop. This loop formation likely increases the accessibility of the cleavage site, and the insertion of critical basic amino acids contributes to these viruses' marked increase in pathogenicity.

## Conclusions

Cleavage of the HA0 protein of influenza viruses results in an essential conformational change in the HA protein, which enables the envelope of the endocytosed virus to fuse with the membrane of endosomes, releasing nucleocapsids into the cytoplasmic compartment (*5**,**13*). The 7-aa insertion described here generates an enlarged exposed loop, which contains multiple basic amino acids in the HA0 protein. Together these modifications to the HA0 protein likely result in an increased rate of cleavage by furinlike proteases, which increases pathogenicity. Analysis of the nucleotide sequences of HA genes from HPAI H5 and H7 isolates has shown that in many cases direct repeats of a purine-rich sequence (AAGAAA) occur. This sequence may arise because of the pausing of the transcriptase complex at a region of secondary structure, which results in slippage of the transcriptase complex and insertion of a short repeat sequence. Additionally, recombination events between the HA and NP genes (*14*) and the HA gene and host cell 28S ribosomal RNA have been documented (*15*). More recently, recombination between the NP and HA genes resulted in a 30-nt insertion near the HA0 cleavage site in HPAI viruses isolated in Chile in 2002 (*16*). The HA sequences from the Fraser Valley outbreak described here contain a novel insert derived from the M gene. Thus, in addition to transcriptase slippage, nonhomologous recombination represents an important mechanism in the acquisition of virulence in avian influenza viruses.

## Appendix

### Materials and Methods

#### Growth of Avian Influenza in Embryonated Chicken Eggs and Cell Culture

The tissue samples were homogenized in 10 volumes of virus isolation diluent (peptone 10 g/L, gelatin 2.5 g/L, phosphate-buffered saline pH 7.4, gentamicin 100 mg/L, amphotericin B 5 mg/L, cyclohexamide 10 mg/L ). The homogenate was centrifuged twice at 3,000 x *g* for 30 min to remove debris, and 0.2 mL of supernatant was injected into four 9- to 11-day-old embryonated eggs obtained from a cloistered nonvaccinated flock. Controls were injected with virus isolation fluid. Eggs were incubated at 37°C and assessed for viability at 24 h. At 48 h, the eggs were cooled, and the allantoic fluid was harvested. A hemagglutination test was run using 0.5% chicken red blood cells, and hemagglutinin-positive samples were confirmed by polymerase chain reaction (PCR). Nasopharyngeal and conjunctival swab specimens from affected patients were injected into primary rhesus monkey kidney cells and passaged further in Madin Darby canine kidney cells (Diagnostic Hybrids, Athens, OH). The isolated viruses were shown to be influenza A H7 by RT (reverse transcription)-PCR of the RNA (*17*) and subsequently typed as H7N3 (National Microbiology Laboratory, Winnipeg, MB, Canada).

### RNA Extraction and RT-PCR

Trizol (Invitrogen, Burlington, ON, Canada) was used to extract vRNA from 100 μL of allantoic fluid from infected embryonated eggs or 1 mL of infected Madin-Darby kidney cell culture. The vRNA was isolated by using Phase Lock Gel, heavy 2 mL Eppendorf tubes (Brinkmann, Mississauga, ON, Canada) according to the manufacturer's recommended protocol. RNA was precipitated in 0.5 volumes of isopropanol in the presence of 0.2 mg of mussel glycogen (Invitrogen, Burlington, ON, Canada). The resulting pellet was washed in 75% ethanol and resuspended in 40 μL of diethylpyrocarbonate-treated dH_2_0. A two step RT-PCR was used to amplify each of the viral gene segments. The RNA (6 μL) was synthesized into cDNA by using 500 ng of Uni12 primer (Table A1) and the Powerscript Reverse Transcriptase (BD Biosciences, Mississauga, ON), according to the manufacturer's recommended protocol. The RT reaction was performed at 42°C for 60 min. The cDNA was amplified by using the Expand Long Template PCR System (Roche Diagnostics, Laval, QB, Canada), following the protocol provided. The Mg^2+^ concentration was 3.5 mM, the primer concentration was 0.4 mmol/L, and betaine was added to a final concentration of 1 mol/L. Segment-specific primers were based on the universal influenza A primer set designed by Hoffman et al. (*18*). The cycling conditions consisted of an initial denaturation at 95^o^C for 5 min followed by 10 touchdown PCR cycles starting with 95°C for 15 s, 68^o^C (decreased by 1^o^C in each subsequent cycle) for 15 s, 68°C for 3 min; then 20 cycles of 95°C for 15 s, 62°C for 15 s, 68°C for 3 min; followed by an extension at 68°C for 10 min.

### Isolation and Cloning of vRNA Gene Segments

Ten microliters of the PCR reaction for each sample was loaded on an 1% agarose gel. The gel was stained with >SYBR Green (Mandel, Guelph, ON, Canada) and visualized using a Typhoon 9400 Variable Mode Imager (Amersham, Baie d'Urfe, QB, Canada). Visible bands of expected size were excised from the agarose gel, and purified using the MinElute Gel Extraction Kit (Qiagen, Mississauga, ON) following the manufacturer's protocol. Purified amplicons were cloned into the pCR4-TOPO vector by using the TOPO TA Cloning Kit for Sequencing (Invitrogen), according to the manufacturer's protocol.

### vRNA gene Segment Sequencing

Eight clone inserts for each genomic segment were randomly selected and sequenced on an ABI PRISM 3730 XL DNA Analyzer with BigDye v3.1 primer cycle sequencing reagents (Applied Biosystems Canada, Streetsville, ON, Canada). End reads were obtained by using T3 and T7 reverse primers. Sequence reads were processed, and their quality was assessed by using *Phred* and assembled by using *Phrap* (*19**,**20*). When needed, specific sequencing primers were designed by manual selection from primer sequences generated by Consed (*21*), and full-length sequences were obtained by primer walking.

The Table A1 gives a comparison of individual gene segments of the Fraser Valley H7N3 outbreak with influenza genes from GenBank showing the highest similarity. Figure A1 provides complete nucleotide and protein alignments for the HA gene for the five isolates sequences in this study, and Figure A2 is a phylogenetic tree of isolates described in the text.

### Homology Modeling

The 0.28-nm resolution crystal structure of the hemagglutinin precursor protein from influenza A virus, human H3, strain CV-1 (PDB code HA01) was used as the template (*22*). An initial sequence alignment was performed with the program ClustalW (*23*) and then modified by hand, resulting in 49.9 % sequence identity (Figure A3). The initial model was made by using the program O (*24*) and then energy minimized with the program CNS (*25*). The monomer model was superimposed onto the trimeric 1HA0 template structure by using the program superimpose (*26*) and then further energy minimized by using CNS. The model includes residues 21 through 519 (lacking the N-terminal 20 residues and the C-terminal 48 residues) of the human A/Canada/504/04 (Hu504) hemagglutinin precursor. The figures were prepared by using the program PyMol (*27*).

## Appendix

**Table A1:** Comparison of individual gene segments of four isolates of the Fraser Valley H7N3 outbreak in 2004 with full length influenza genes from GenBank showing the highest similarity^a^

Query accession no.	Isolate name	Length (bp)	BLASTN identity (%)	Accession no. and isolate name	BLASTP identity (%)	Accession no. and isolate name
AY650270	A/Chicken/Canada/AVFV1/04 HA	1,733	95	AF072384 A/Chicken/NewYork/13142-5/94 (H7N3)	97	AAD26923 A/Chicken/NewYork/13142-5/94 (H7N3)
AY648287	A/Chicken/Canada/AVFV2/04 HA	1,754	96	AF072384 A/Chicken/NewYork/13142-5/94 (H7N3)	96	AAD26923 A/Chicken/NewYork/13142-5/94 (H7N3)
AY611524	A/Canada/444/04 (human) HA	1,754	96	AF072384 A/Chicken/NewYork/13142-5/94 (H7N3)	96	AAD26923 A/Chicken/NewYork/13142-5/94 (H7N3)
AY646078	A/Canada/504/04 (human) HA	1,754	96	AF072384 A/Chicken/NewYork/13142-5/94 (H7N3)	96	AAD26923 A/Chicken/NewYork/13142-5/94 (H7N3)
AY650271	A/Chicken/Canada/AVFV1/04 M	1,027	98	AF073198 A/Turkey/Colorado/13356/91(H7N3NSA)	100 (M1)	AAB19773 A/Turkey/Minnesota/833/80 (H4N2)
					100 (M2)	AAQ77433 A/Chicken/Chile/176822/02 (H7N3)
AY648288	A/Chicken/Canada/AVFV2/04 M	1,027	98	AF073198 A/Turkey/Colorado/13356/91(H7N3NSA)	100 (M1)	AAB19773
					100 (M2)	AAQ77433
AY611525	A/Canada/444/04 (human) M	1,027	98	AF073198 A/Turkey/Colorado/13356/91(H7N3NSA)	100 (M1)	AAB19773
					100 (M2)	AAQ77433
AY646079	A/Canada/504/04 (human) M	1,027	98	AF073198 A/Turkey/Colorado/13356/91(H7N3NSA)	100 (M1)	AAB19773
					100 (M2)	AAQ77433
AY650272	A/Chicken/Canada/AVFV1/04 NA	1,453	97	AY300951 A/blue-winged teal/TX/2/01 (H7N3)	98	AAP57563 A/blue-winged teal/TX/2/01 (H7N3)
AY648289	A/Chicken/Canada/AVFV2/04 NA	1,453	98	AY300951 A/blue-winged teal/TX/2/01 (H7N3)	98	"
AY611526	A/Canada/444/04 (human) NA	1,453	97	AY300951 A/blue-winged teal/TX/2/01 (H7N3)	97	"
AY646080	A/Canada/504/04 (human) NA	1,453	98	AY300951 A/blue-winged teal/TX/2/01 (H7N3)	98	"
AY650273	A/Chicken/Canada/AVFV1/04 NP	1,565	95	M30766 A/ruddyturnstone/NewJersey/47/85 (H4N6)	100	AAG17432 A/Swine/Ontario/)1911-1/99 (H4N6)
AY648290	A/Chicken/Canada/AVFV2/04 NP	1,565	95%	M30766 A/ruddyturnstone/NewJersey/47/85 (H4N6)	99	"
AY611527	A/Canada/444/04 (human) NP	1,565	95%	M30766 A/ruddyturnstone/NewJersey/47/85 (H4N6)	99	"
AY646081	A/Canada/504/04 (human) NP	1,565	95%	M30766 A/ruddyturnstone/NewJersey/47/85 (H4N6)	99	"
AY650274	A/Chicken/Canada/AVFV1/04 NS	890	98%	U85391 A/Turkey/Minnesota/10734/95	99 (NS1)	AAA43130 A/Anas acuta/Primrje/695/76 (H3N2)
					99 (NS2)	AAK14369 A/Brevig-Mission/1/18 (H1N1)
AY648291	A/Chicken/Canada/AVFV2/04 NS	890	98	U85391 A/Turkey/Minnesota/10734/95	99 (NS1)	AAA43130
					99 (NS2)	AAK14369
AY611528	A/Canada/444/04 (human) NS	890	98	U85391 A/Turkey/Minnesota/10734/95	99 (NS1)	AAA43130
					99 (NS2)	AAK14369
AY646082	A/Canada/504/04 (human) NS	890	98	U85391 A/Turkey/Minnesota/10734/95	99 (NS1)	AAA43130
AY650275	A/Chicken/Canada/AVFV1/04 PA	2,230	94	AF285890 A/Swine/Ontario/01011-1/99 (H4N6)	98	CAB56290 A/Mallard.NewYork/6750/78
AY648292	A/Chicken/Canada/AVFV2/04 PA	2,233	95	AF285890 A/Swine/Ontario/01011-1/99 (H4N6)	98	CAB56290 A/Mallard.NewYork/6750/78
AY616764	A/Canada/444/04 (human) PA	2,233	95	AF285890 A/Swine/Ontario/01011-1/99 (H4N6)	98	CAB56290 A/Mallard.NewYork/6750/78
AY646083	A/Canada/504/04 (human) PA	2,233	95	AF285890 A/Swine/Ontario/01011-1/99 (H4N6)	98	CAB56290 A/Mallard.NewYork/6750/78
AY653039	A/Chicken/Canada/AVFV1/04 PB1	2,341	95	M25925 A/Turkey/Minnesota/833/80 (H4N2)	99	AAG17436 A/Swine/Ontarion/01911-1/99 (H4N6)
AY648293	A/Chicken/Canada/AVFV2/04 PB1	2,341	95	M25925 A/Turkey/Minnesota/833/80 (H4N2)	99	AAG17436 A/Swine/Ontarion/01911-1/99 (H4N6)
AY616765	A/Canada/444/04 (human) PB1	2,341	95	M25925 A/Turkey/Minnesota/833/80 (H4N2)	98	AAG17436 A/Swine/Ontarion/01911-1/99 (H4N6)
AY646084	A/Canada/504/04 (human) PB1	2,341	95	M25925 A/Turkey/Minnesota/833/80 (H4N2)	99	AAG17436 A/Swine/Ontarion/01911-1/99 (H4N6)
AY650276	A/Chicken/Canada/AVFV1/04 PB2	2,341	93	M73522 A/Seal/Massachusetts/133/82 (H4N5)	99	AAR04359 A/Chicken/Netherlands/1/03 (H7N7)
AY648294	A/Chicken/Canada/AVFV2/04 PB2	2,341	93	M73522 A/Seal/Massachusetts/133/82 (H4N5)	99	AAR04359 A/Chicken/Netherlands/1/03 (H7N7)
AY616766	A/Canada/444/04 (human) PB2	2,341	93	M73522 A/Seal/Massachusetts/133/82 (H4N5)	99	AAR04359 A/Chicken/Netherlands/1/03 (H7N7)
AY646085	A/Canada/504/04 (human) PB2	2,341	93	M73522 A/Seal/Massachusetts/133/82 (H4N5)	99	AAR04359 A/Chicken/Netherlands/1/03 (H7N7)

^a^Query accession no., isolate name and length (bp); GenBank accession no., isolate name, and nucleotide length for each gene segment, respectively; BLASTN identity, percentage of identical nucleotides as assigned by BLASTN; accession no., GenBank nucleotide accession no., and isolate name; BLASTP identity, percentage of identical nucleotides as assigned by BLASTP; accession no., GenBank protein accession no., and isolate name.
